# Genetic modifiers of muscular dystrophy act on sarcolemmal resealing and recovery from injury

**DOI:** 10.1371/journal.pgen.1007070

**Published:** 2017-10-24

**Authors:** Mattia Quattrocelli, Joanna Capote, Joyce C. Ohiri, James L. Warner, Andy H. Vo, Judy U. Earley, Michele Hadhazy, Alexis R. Demonbreun, Melissa J. Spencer, Elizabeth M. McNally

**Affiliations:** 1 Center for Genetic Medicine, Northwestern University Feinberg School of Medicine, Chicago, Illinois, United States of America; 2 Department of Neurology, David Geffen School of Medicine at UCLA, Los Angeles, California, United States of America; 3 Committee on Development, Regeneration, and Stem Cell Biology, The University of Chicago, Chicago, Illinois, United States of America; The Jackson Laboratory, UNITED STATES

## Abstract

Genetic disruption of the dystrophin complex produces muscular dystrophy characterized by a fragile muscle plasma membrane leading to excessive muscle degeneration. Two genetic modifiers of Duchenne Muscular Dystrophy implicate the transforming growth factor β (TGFβ) pathway, osteopontin encoded by the *SPP1* gene and latent TGFβ binding protein 4 (*LTBP4*). We now evaluated the functional effect of these modifiers in the context of muscle injury and repair to elucidate their mechanisms of action. We found that excess osteopontin exacerbated sarcolemmal injury, and correspondingly, that loss of osteopontin reduced injury extent both in isolated myofibers and in muscle in vivo. We found that ablation of osteopontin was associated with reduced expression of TGFβ and TGFβ-associated pathways. We identified that increased TGFβ resulted in reduced expression of *Anxa1* and *Anxa6*, genes encoding key components of the muscle sarcolemma resealing process. Genetic manipulation of *Ltbp4* in dystrophic muscle also directly modulated sarcolemmal resealing, and *Ltbp4* alleles acted in concert with *Anxa6*, a distinct modifier of muscular dystrophy. These data provide a model in which a feed forward loop of TGFβ and osteopontin directly impacts the capacity of muscle to recover from injury, and identifies an intersection of genetic modifiers on muscular dystrophy.

## Author summary

Genetic modifiers for muscular dystrophy have been identified through transcriptomic and genomic profiling in humans and mouse models. Two modifiers, *Ltbp4* and *Spp1*, encode extracellular proteins while a third modifier, *Anxa6*, specifies a membrane-associated protein. Using a model of muscle injury, we assessed the interaction of these modifiers, identifying a feed forward loop between *Ltbp4* and *Spp1* that promotes TGFβ signaling. This feed forward loop is expected to contribute to the progressive nature of muscular dystrophy. We also evaluated the interaction between *Anxa6* and *Ltbp4*, identifying an additive effect of these two genetic modifiers.

## Introduction

Muscular dystrophies are inherited diseases that cause progressive muscle wasting [1]. Many muscular dystrophies are caused by mutations in genes encoding for components of the dystrophin glycoprotein complex, which anchors the actin cytoskeleton of myofibers to their cell membrane, the sarcolemma. Loss of function mutations in the *DMD* gene cause Duchenne muscular dystrophy (DMD), while mutations in the *SGCG* gene, which encodes the dystrophin associated protein γ-sarcoglycan, cause limb-girdle muscular dystrophy type 2C (LGMD 2C) [2, 3]. Disruption of the dystrophin complex results in loss of membrane integrity, leading to chronic injury and necrosis of myofibers [4]. Detrimental remodeling, with replacement by fibrofatty tissue, leads to ongoing, progressive impairment of muscle function [1]. This pathological process begins with disruption of the sarcolemma, and mechanisms to enhance sarcolemmal repair may provide insight in possible therapeutic targets for treating muscular dystrophy.

Disease progression in the muscular dystrophies is variable even in the presence of the same primary mutation, suggesting that secondary genetic variants, or genetic modifiers, can considerably impact the outcome of muscle wasting [5]. The effect of modifiers is evident in murine models of muscular dystrophy, where the same genetic mutation results in significantly different outcomes dependent on the genetic background of the mouse strain [6]. Dystrophin deficiency is modeled in mice by the *mdx* mutation, a premature stop codon in exon 23 of the dystrophin gene, while γ-sarcoglycan deficiency is modeled by mice lacking exon 2 of the *Sgcg* gene [3, 7]. *mdx* and *Sgcg* mutations have been shown to cause muscular dystrophy with strain-dependent variable pathology, which is severe in the DBA/2J genetic background, intermediate in the C57/Bl6-Bl10 strains, and more mild in the 129T2/SvEmsJ (129T2) background [6, 8, 9].

Identification of genetic modifiers and their mechanisms of action is a useful approach to refine prognosis and potentially discover novel therapeutic targets. Several candidate modifiers act as extracellular agonists of signaling cascades, including osteopontin, encoded by the *SPP1* gene, and latent TGFβ binding protein 4 (*LTBP4*). Osteopontin is a secreted glycoprotein that signals through integrin and CD44 receptors [10]. *SPP1* expression is highly upregulated in affected muscles of humans and animals with muscular dystrophy [11–19]. Genetic loss of *Spp1* in *mdx* mice correlates with greater strength, less fibrosis and milder pathology, as compared to control *mdx* littermates [13]. Moreover, *Spp1* ablation has been linked to a shift in macrophage polarization towards a regenerative phenotype in *mdx* muscles [20]. In humans with DMD, a single nucleotide polymorphism (SNP) in the *SPP1* promoter (GG/TG) correlates with increased grip strength and later loss of ambulation compared to patients with the more prevalent SNP (TT), especially in DMD individuals who are steroid treated [19, 21, 22]. Some genetic cohort studies have not shown this same effect [23, 24]. The manner in which the *SPP1* SNP affects osteopontin expression with disease progression is complex, and it is unclear in which *SPP1*-expressing cell type(s) this modifier SNP acts.

*Ltbp4* was identified as genetic modifier of several pathologic traits in the *Sgcg* mouse model, including sarcolemmal damage and fibrosis [25]. Latent TGFβ binding protein (LTBP4) is an extracellular protein that binds TGFβ, releasing it upon proteolysis of its hinge region [26]. The LTBP4 modifier also correlates with differential outcomes in humans with muscular dystrophy [21, 23, 24]. In mice, the “risk” *Ltbp4* allele encodes a shorter hinge region that is more susceptible to proteolysis, and this risk allele is found in the DBA/2J strain correlating with more severe muscular dystrophy. In contrast, most mouse strains including 129T2 and C57 substrains have the protective *Ltbp4* allele encoding a longer hinge region that is more resistant to proteolytic cleavage. Overexpression of the protective *Ltbp4* allele in the *mdx* mouse reduces fibrosis and promotes muscle growth [26]. However, the specific molecular effects of protective and deleterious *Ltbp4* alleles on sarcolemmal resealing and repair are still unknown.

Intriguingly, it has been shown that the *Spp1* promoter is susceptible to TGFβ-driven transcriptional activation [27, 28]. In mesenchymal cells, osteopontin signaling activates *Tgfb1* transcription via the myeloid zinc finger 1 (MZF1) transcriptional factor [29, 30]. These data support a potential interaction between these two modifiers, *Spp1* and *Ltbp4*, in muscular dystrophy. Examining the combinatorial effects of genetic modifiers requires a large population, which is challenging for a rare human disorder like DMD. Here we asked whether genetic manipulation of osteopontin and *Ltbp4* impacts sarcolemmal resealing and repair. To specifically address this question, we relied on optimized conditions of sarcolemmal micro-injury, and real-time detection of sarcolemmal damage and repair cap formation in isolated live myofibers [31, 32]. Laser-mediated injury results in larger sarcolemmal damage in dystrophic myofibers than in wildtype myofibers using age- and background-matched conditions [33]. We found that both osteopontin and *Ltbp4* modify sarcolemmal repair in wildtype and dystrophic myofibers. Furthermore, we documented how osteopontin and the deleterious *Ltbp4* isoform converge in a feed-forward TGFβ signaling loop that correlated with transcriptional repression of annexin genes and impaired sarcolemmal repair. In addition, we dissected the relative impact of the different alleles of *Ltbp4* and a third modifier *Anxa6* on resealing and repair of injured sarcolemma in background-strain matched conditions. These results indicate that osteopontin and LTBP4 regulate a TGFβ signaling loop to modify sarcolemmal repair in normal and dystrophic muscle.

## Results

### Recombinant osteopontin worsens sarcolemmal injury

**Fig 1:**
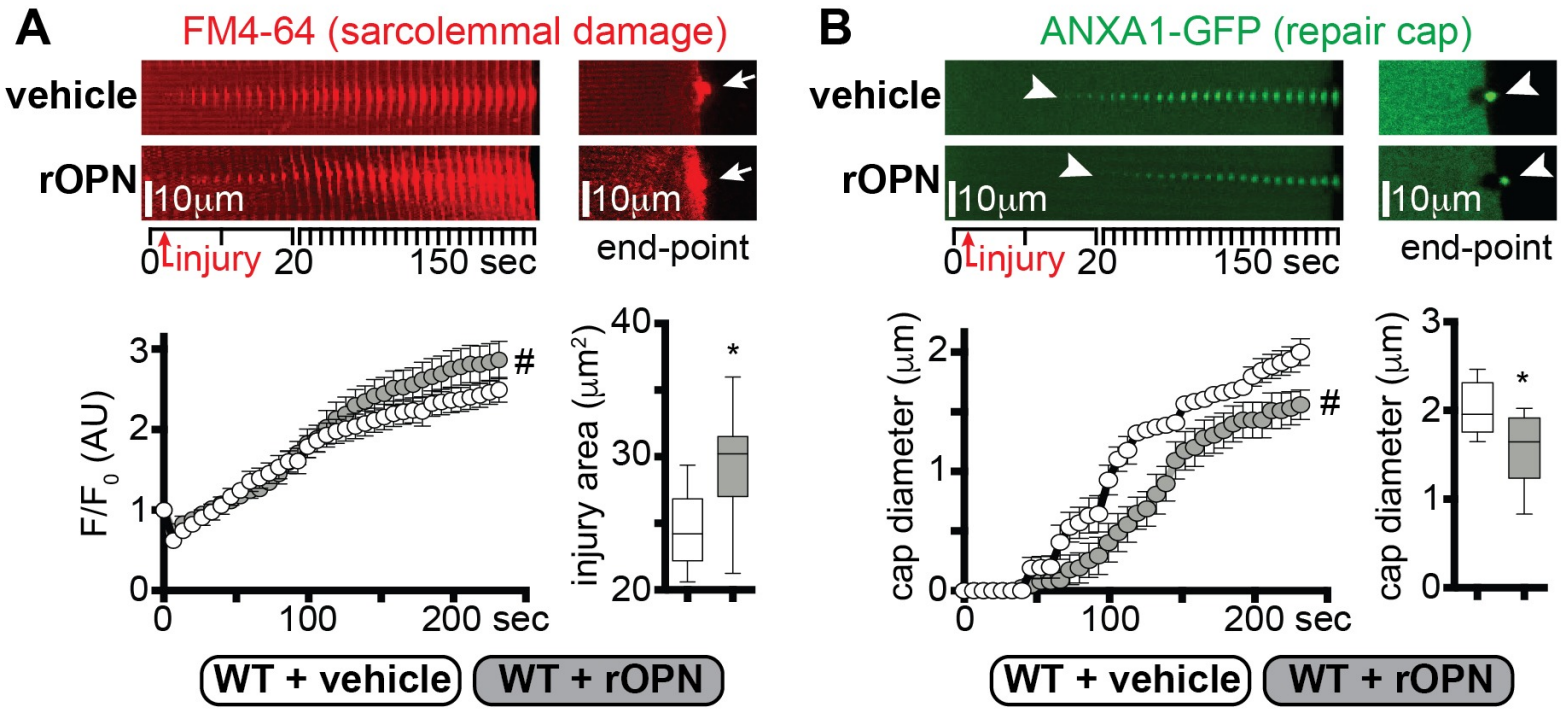
Excess osteopontin exacerbates sarcolemmal injury and delays repair cap formation. The sarcolemmal wounding assay was performed on *flexor digitorus brevis* muscles from WT muscle in the presence or absence of recombinant osteopontin (rOPN; 10μl at 1μg/μl). **A)** The extent of sarcolemmal damage, monitored as FM4-64 area, was greater in rOPN-treated myofibers compared to vehicle-treated control, both over time and at end-point (arrows). **B)** Repair cap formation, monitored by GFP-tagged annexin A1 (GFP-ANXA1) was delayed in rOPN-treated myofibers (arrowheads). Repair cap diameter at end-point was smaller in treated compared to control myofibers (arrowheads). Over time image series represents stacked consecutive images of the injured site at 10° orientation to reveal the extent of dye accumulation or repair cap formation (S1B Fig). FM4-64 and ANXA1-GFP pictures were acquired simultaneously. Marked line plots, avg±sem; box plots, Tukey distribution; n = 50 myofibers (5 mice)/group; #, P<0.05 vs vehicle, 2way ANOVA + Bonferroni; *, P<0.05 vs vehicle, unpaired t-test with Welch’s correction.

To gain insight in the role of osteopontin (OPN) in myofiber repair, we assessed the effect of recombinant osteopontin (rOPN) on sarcolemmal repair of isolated myofibers [32]. We tested the repair capacity after laser-mediated sarcolemmal injury and quantified two parameters in real time: (i) accumulation of the FM4-64 dye at the injury site, marking the extent of damage; (ii) formation of the repair complex monitoring GFP-tagged annexin A1 (ANXA1-GFP) protein [31]. *DBA/2J* wildtype (WT) mice were injected intramuscularly with 10μg rOPN at 1, 3 and 5 days after electroporation with the plasmid encoding ANXA1-GFP. The activity of injected rOPN was confirmed by monitoring expression of *Mmp2* and *Mzf1*, factors known to be downstream of OPN [30, 34]. *Mmp2* and *Mzf1* were increased 48 hours after rOPN injection while vehicle injection did not stimulate this response (S1A Fig). Seven days after electroporation, live myofibers from treated and vehicle injected control muscles were subjected to laser injury. FM4-64 accumulation, which marks the area of injury, was greater in rOPN injected muscle at 240 seconds after laser injury, as compared to vehicle-treated myofibers (Fig 1A). Moreover, the onset of the annexin A1 repair cap formation was significantly slower in rOPN-injected muscle than control myofibers, resulting in a significantly smaller annexin A1 cap size at end-point (Fig 1B). Thus, intramuscular injection of *DBA/2J* WT muscle with rOPN exacerbated sarcolemmal injury and slowed formation of the annexin-containing repair complex.

### Genetic ablation of osteopontin modifies sarcolemmal injury of dystrophic myofibers

**Fig 2:**
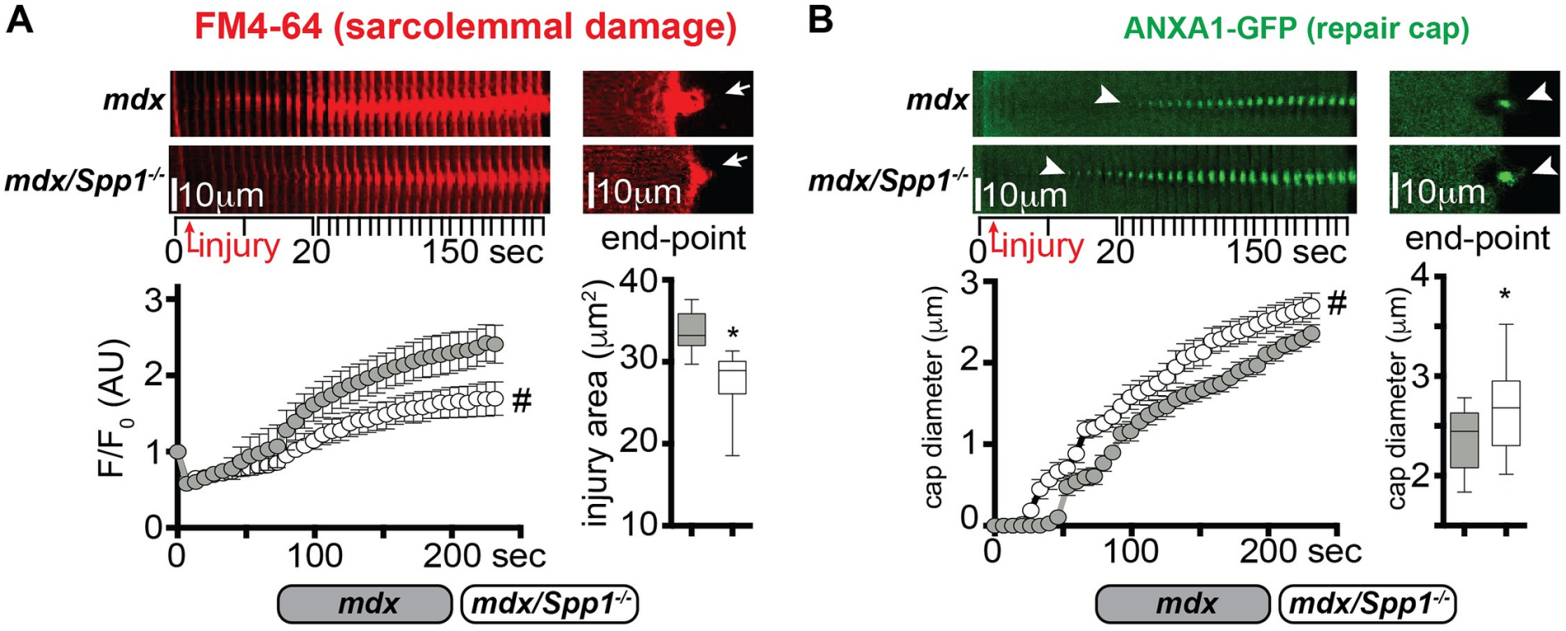
*mdx* mice lacking osteopontin (mdx/*Spp1*^*-/-*^*)* recover from sarcolemmal injury more efficiently. **A)** The sarcolemmal wounding assay was performed on *flexor digitorum brevis* myofibers from *mdx* or *mdx/Spp1*^*-/-*^ animals, after electroporation with the ANXA1-GFP-encoding plasmid. The extent of sarcolemmal damage, monitored through accumulation of FM4-64 dye after laser injury, was significantly reduced in *mdx*/*Spp1*^*-/-*^ myofibers compared to control dystrophic myofibers. This was quantified over time as dye accumulation at site of injury (left) and area of injury at end-point (right; arrows). The time series (left images) represent stacked consecutive images of the injured site at 10° orientation to reveal the extent of dye accumulation. **B)** Repair cap onset, monitored by GFP-tagged ANXA1, was significantly faster in *mdx/Spp1*^*-/-*^ myofibers (arrowheads), resulting in a larger cap diameter at end-point (arrowheads). Time series of stacked consecutive images of the injured site at 10° orientation revealed the extent of dye accumulation or repair cap formation. FM4-64 and ANXA1-GFP pictures were acquired simultaneously. Marked line plots, avg±sem; box plots, Tukey distribution; n = 40 myofibers (4 mice)/group; #, P<0.05 vs *mdx* control, 2way ANOVA + Bonferroni; *, P<0.05 vs *mdx* control, unpaired t-test with Welch’s correction.

Genetic ablation of *Spp1* in *mdx* mice results in decreased fibrosis and muscle pathophysiology in *mdx* mice [13]. We asked whether *Spp1* loss had a measurable effect on sarcolemmal repair of *mdx* myofibers. To this end, we compared myofibers from 20 week-old *mdx* (*Spp1*^*+/+*^; control animals) and age-matched *Spp1*-deficient *mdx* (*mdx/Spp1*^*-/-*^) littermates for the extent of sarcolemmal damage after laser injury. Myofibers from both mice groups were electroporated with the ANXA1-GFP-encoding plasmid prior to harvest and laser-injury assay. *Spp1*-deficient dystrophic myofibers showed significantly reduced FM4-64 accumulation at the injury site over time, and a reduction of injury area at end-point when compared to control myofibers (Fig 2A). We monitored annexin A1 repair cap appearance at the site of injury and observed faster rate of cap appearance in *mdx/Spp1*^*-/-*^ compared to *mdx* control myofibers, resulting in a larger cap size at analysis end-point (Fig 2B). Furthermore, expression levels of endogenous *Anxa1* and *Anxa6*, encoding the repair cap proteins annexins A1 and A6, were significantly upregulated in muscle of *mdx/Spp1*^*-/-*^ mice, as compared to littermate control animals (S2A Fig). Moreover, improved sarcolemmal repair in isolated fibers was also reflected in *in vivo* findings in muscle. *Spp1* deficiency correlated with lower levels of fibrosis, quantified as hydroxyproline content, in both quadriceps and diaphragm muscles, as well as with reduced levels of circulating creatine kinase, a marker of striated muscle damage (S2B and S2C Fig), consistent with previous characterization of *mdx/Spp1*^*-/-*^ mice [13] [20].

**Fig 3:**
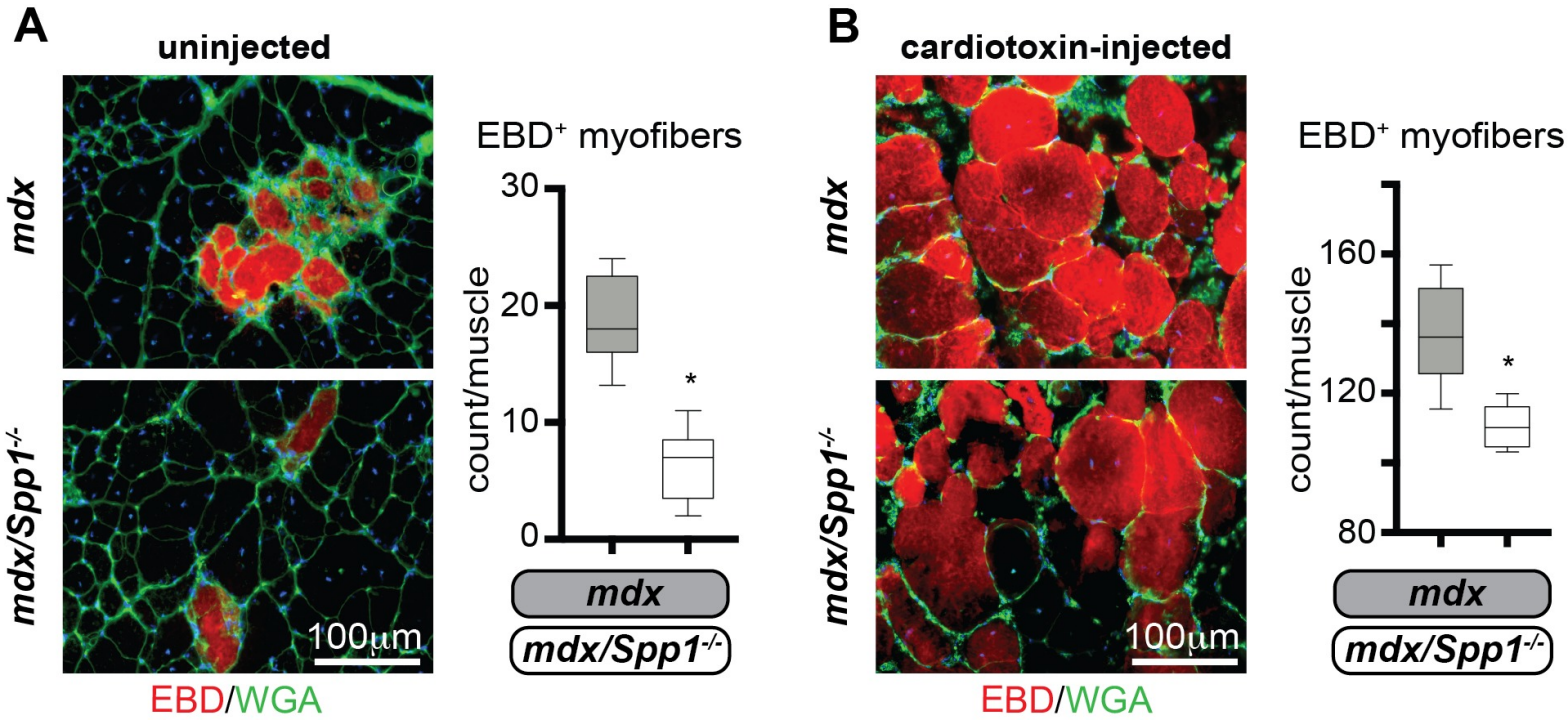
*Spp1* ablation in *mdx* muscle resulted in fewer disrupted myofibers in vivo. **A)** Three hours after systemic EBD delivery, the number of EBD-positive myofibers (red appearance) was significantly less in *mdx/Spp1*^*-/-*^ TA muscles than in control *mdx* muscles (left, representative immunofluorescence panels; right, quantitation). **B)** Cardiotoxin injection was performed in the contralateral muscles, increasing the number of EBD-positive fibers. After cardiotoxin injury, there were fewer EBD-positive myofibers in *mdx/Spp1*^*-/-*^ compared to *mdx* muscles (left, representative immunofluorescence panels; right, quantitation). Box plots, Tukey distribution; n = 4 mice/group; *, P<0.05 vs *mdx* control, unpaired t-test with Welch’s correction.

In addition to *Spp1’s* role in dystrophic muscle remodeling, osteopontin exacerbates injury in toxin-injured wildtype muscles [35]. To assess *Spp1’s* role in chronic and acute injury of dystrophic muscle, the *tibialis anterior* (TA) muscles of *mdx* and *mdx/Spp1*^*-/-*^ mice were injected with cardiotoxin along the muscle axis, immediately after systemic delivery of Evans Blue Dye (EBD). Each mouse received cardiotoxin in one muscle, while the contralateral remained uninjected. After three hours, muscles were harvested and injury extent was quantified as EBD-positive myofibers per muscle using serial cross-sections. There were fewer EBD-positive myofibers in *mdx/Spp1*^*-/-*^ muscles than in *mdx* muscles, both under basal conditions and after toxin-induced injury (Fig 3A and 3B). Thus, these data suggest that sarcolemmal repair is enhanced and muscle injury is reduced after genetic ablation of osteopontin.

### *Spp1* deletion correlates with decreased TGFβ signaling in dystrophic muscle

**Fig 4:**
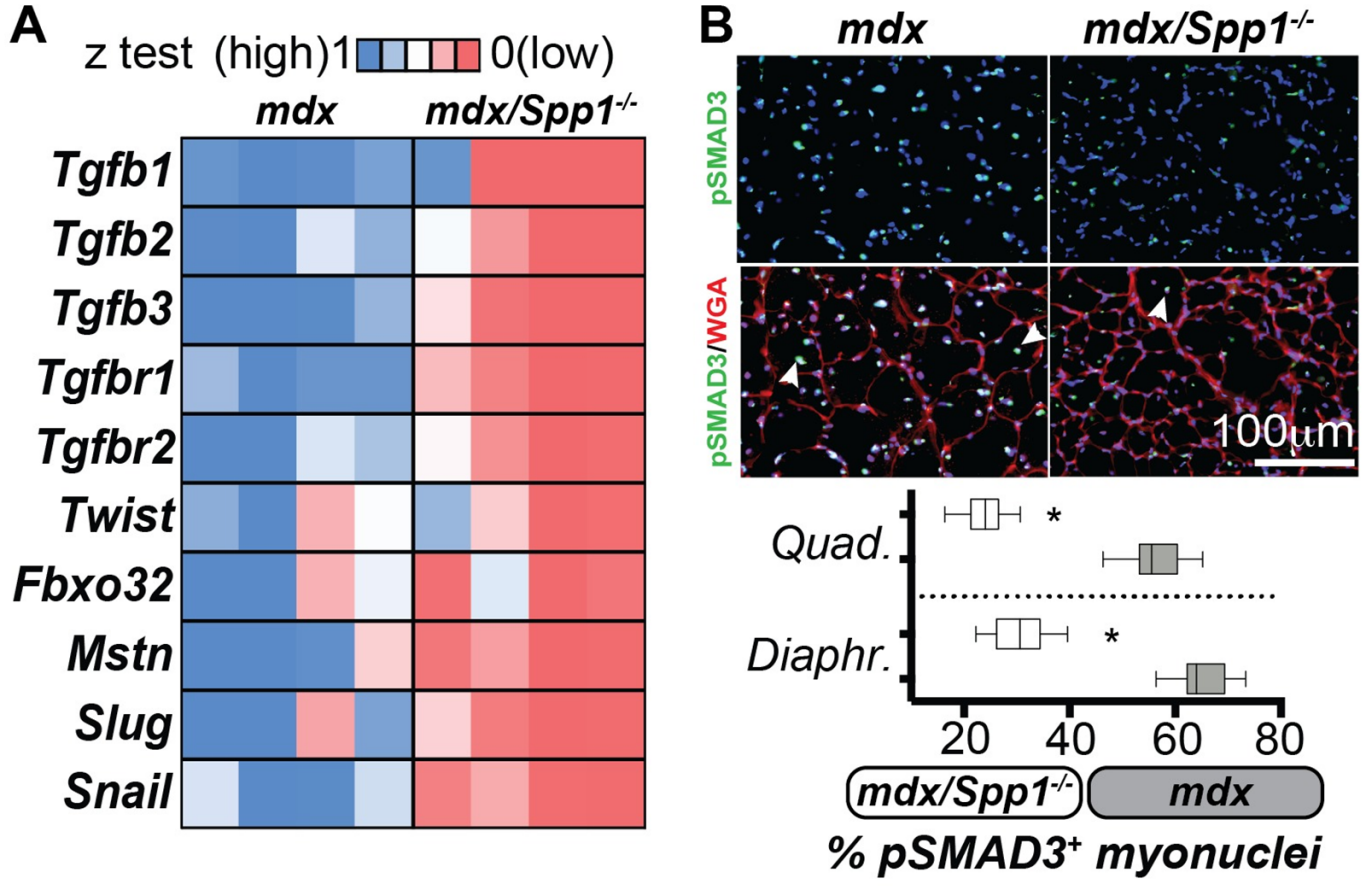
*Spp1* ablation in *mdx* muscle is associated with decreased TGFβ gene expression and signaling. **A)** Heat-map of qPCR analysis of gene expression, normalized to control muscles, showed downregulation of genes associated with the TGFβ pathway and atrophic remodeling in *mdx*/*Spp1*^*-/-*^ dystrophic muscle (*tibialis anterior*). All genes shown in the heatmap were significantly different (*). **B)** Percentage of phosphorylated-Smad3-positive nuclei within myofibers (myonuclei; arrowheads) was significantly decreased in *quadriceps* (representative immunofluorescence panels) and *diaphragm* muscles of *mdx/Spp1*^*-/-*^ mice when compared to *mdx* mice. Box plots, Tukey distribution; heat-map, z-test average values of fold change to control (ctrl); n = 4 mice/group; *, P<0.05 vs *mdx* control, 1way ANOVA + Bonferroni.

The *Spp1* promoter is responsive to TGFβ signaling, and the TGFβ1 ligand increases *Spp1* levels [27, 28]. We therefore asked whether TGFβ signaling was altered in *mdx/Spp1*^*-/-*^ muscles. To this end, we compared *mdx/Spp1*^*-/-*^ and control *mdx* muscles for expression levels of TGFβ pathway genes and for enrichment of myonuclei positive for phosphorylated SMAD3 (pSMAD3), a known effector of active TGFβ signaling [36]. Quantitative PCR (qPCR) analysis of TA muscles showed that ligands, receptors and downstream factors of the TGFβ pathway, including transcriptional repressors *Slug* and *Snail* [37, 38], were significantly downregulated in *mdx/Spp1*^*-/-*^ mice as compared to *mdx* (Fig 4A). Immunofluorescence microscopy (IFM) of *quadriceps* and *diaphragm* muscles showed that the relative ratio of pSMAD3^+^ myonuclei was significantly reduced in both muscles of *mdx/Spp1*^*-/-*^ mice compared to *mdx* control animals (Fig 4B). Thus, genetic loss of *Spp1* correlated with decreased TGFβ signaling in multiple *mdx* muscles.

### Osteopontin decreases annexin gene expression via TGFβ pathway stimulation

**Fig 5:**
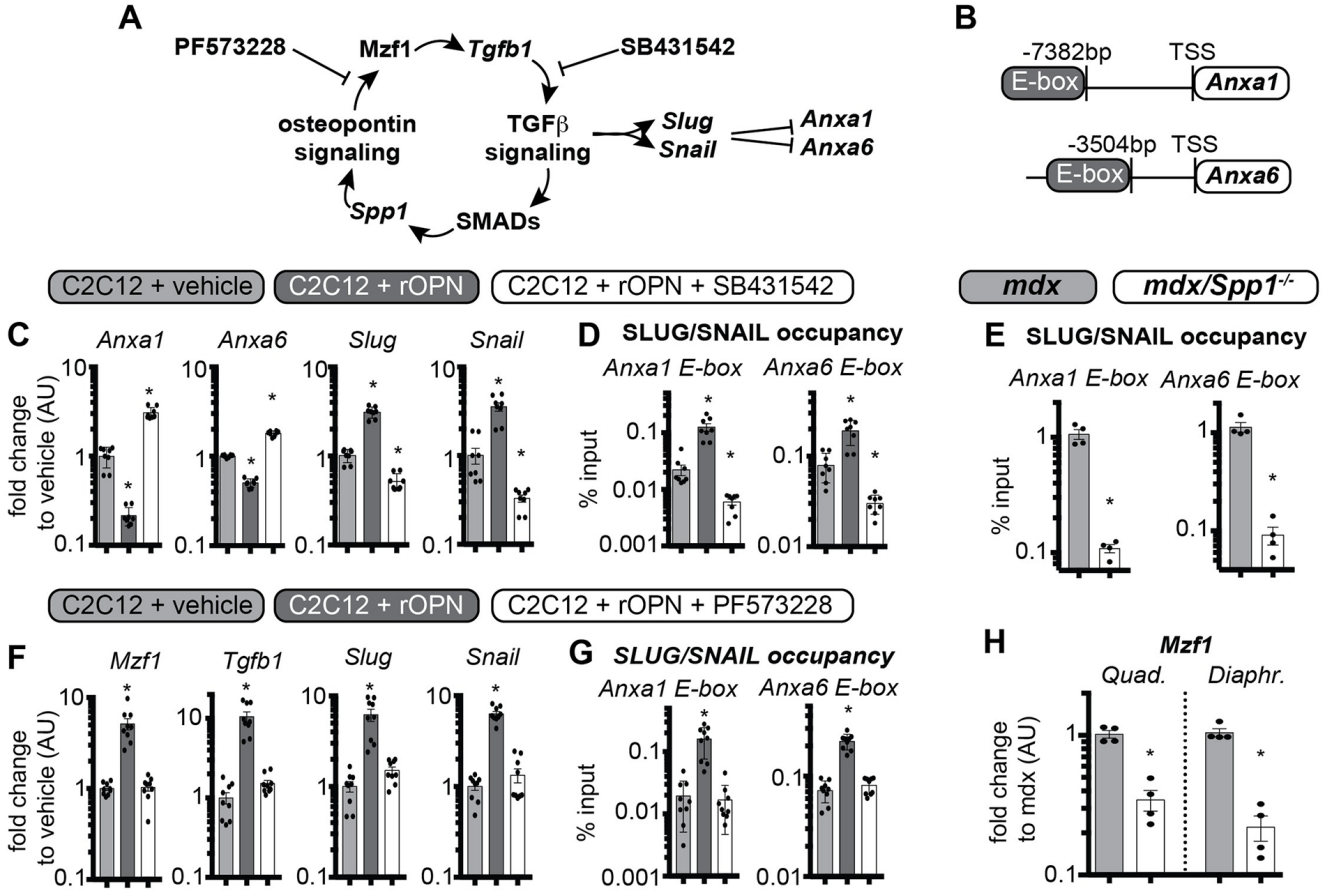
Excess osteopontin and TGFβ activation inhibits *Anxa1* and *Anxa6* gene expression. **A)** Model depicting the feed-forward loop in which osteopontin promotes TGFβ, which in turn promotes *Spp1*/osteopontin expression. *Slug/Snail* expression, downstream of TGFβ activation, results in *Anxa1/Anxa6* gene repression. **B)** Diagram of putative E-box elements upstream of transcriptional start site (TSS) of *Anxa1* and *Anxa6* genes in the murine genome. **C)** qPCR analysis of C2C12 cells after treatment with recombinant osteopontin (rOPN) or rOPN plus the TGFβ inhibitor SB431452. rOPN increased *Slug/Snail* expression, and this was reversed in the presence of SB431452. **D)** Correspondingly, ChIP-qPCR showed that the E-box occupancy of the *Anxa1* and *Anxa6* promoters by the SLUG/SNAIL protein complex was increased by rOPN, and this was reversed in the presence of SB431452. **E)** *Anxa1 and Anxa6* promoter occupancy by SLUG/SNAIL was decreased in the myofiber fraction of *mdx/Spp1*^*-/-*^ muscle compared to control *mdx* muscle (*gastrocnemius*). **F)** PF573228, a chemical inhibitor of OPN-driven signaling leading to *Mzf1-Tgfb1* axis activation, ablated rOPN-induced upregulation of *Slug*, *Snail*, *Mzf1*, and *Tgfb1*. **G)** Accordingly, PF573228 blunted rOPN-dependent increase in SLUG/SNAIL occupancy on *Anxa1/6* E-boxes in C2C12 myoblasts. **H)** *Mzf1*, transcriptional regulator linking the OPN and TGFβ signaling cascades, was downregulated in both hindlimb (*quadriceps*) and respiratory (*diaphragm*) muscles of *mdx/Spp1*^*-/-*^ compared to *mdx*. Histograms, single values & avg±sem; n = 8 independent replicates/group for C2C12 myoblast analyses, n = 4 mice/group for *mdx*/*Spp1*^*-/-*^ mice analyses; (C-D; E-G) *, P<0.05 vs vehicle, 1way ANOVA + Bonferroni; (D) *, P<0.05 vs *mdx* control, unpaired t-test with Welch’s correction.

In non-muscle mesenchymal cells, OPN has been shown to stimulate *TGFβ1* upregulation via integrin signaling and the transcriptional factor *Mzf1* [29, 30]. We hypothesized a feed-forward model, in which OPN sustains TGFβ signaling in muscle and its downstream transcriptional factors *Slug* and *Snail*, which bind E-box DNA elements of target genes (Fig 5A) [39]. Through sequence alignment, we identified a predicted E-box element (GTCGAC motif) [39] upstream of the transcriptional start site of *Anxa1* (-7382bp) and *Anxa6* genes (-3504bp) (Fig 5B). We tested this pathway in C2C12 myoblasts as well as in the myofiber fraction from *mdx* muscles using qPCR and chromatin immunoprecipitation (ChIP). C2C12 cells were exposed to 1μg/ml rOPN, and after 48 hours of rOPN treatment, both *Anxa1* and *Anxa6* were downregulated, while *Slug* and *Snail* were upregulated, as compared to vehicle-treated cells. These responses were reversed when C2C12 cells were co-treated with rOPN along with 10μM SB431542, a chemical compound specifically inhibiting TGFβ signaling activation (Fig 5C) [40]. ChIP-qPCR analysis showed that occupancy of *Anxa1* and *Anxa6* E-box elements by the SLUG/SNAIL transcriptional complex was increased after rOPN treatment, but decreased in the presence of TGFβ inhibitor (Fig 5D). In the myofiber fractions from *mdx/Spp1*^*-/-*^ muscle where *Anxa1* and *Anxa6* expression were increased, these promoters had reduced occupancy of the E-box elements when compared to myofibers from *mdx* littermates (Fig 5E). When C2C12 cells were co-treated with rOPN and 10μM PF573228, a chemical inhibitor of the OPN-driven signaling leading to *Mzf1-Tgfb1* axis activation [41], there was blunting of rOPN-associated upregulation of *Mzf1*, *Tgfb1*, *Slug*, and *Snail* (Fig 5F). Consistent with this, SLUG/SNAIL occupancy of *Anxa1/6* E-box elements reverted to vehicle-like levels when cells were co-treated with rOPN and PF573228 (Fig 5G). Finally, expression of *Mzf1* was significantly lower in *Spp1-*deficient hindlimb and respiratory muscles than in control *mdx* muscles (Fig 5H). Thus, excess OPN is associated with repression of *Anxa1* and *Anxa6*, and increased occupancy of their putative E-box elements by the transcriptional repressor complex SLUG/SNAIL. Repression by SLUG/SNAIL was associated with increased *Mzf1* and *Tgfb1* levels. Moreover, OPN-associated events were reversed in the presence of a chemical TGFβ inhibitor and were blunted by a chemical inhibitor of OPN signaling, thus supporting the hypothesis of a feed-forward circuitry that involves osteopontin and TGFβ pathways suppressing *Anxa1* and *Anxa6* and impairing sarcolemmal repair.

### The DBA/2J genetic background is associated with higher levels of osteopontin and TGFβ signaling, and increased sarcolemmal damage

**Fig 6:**
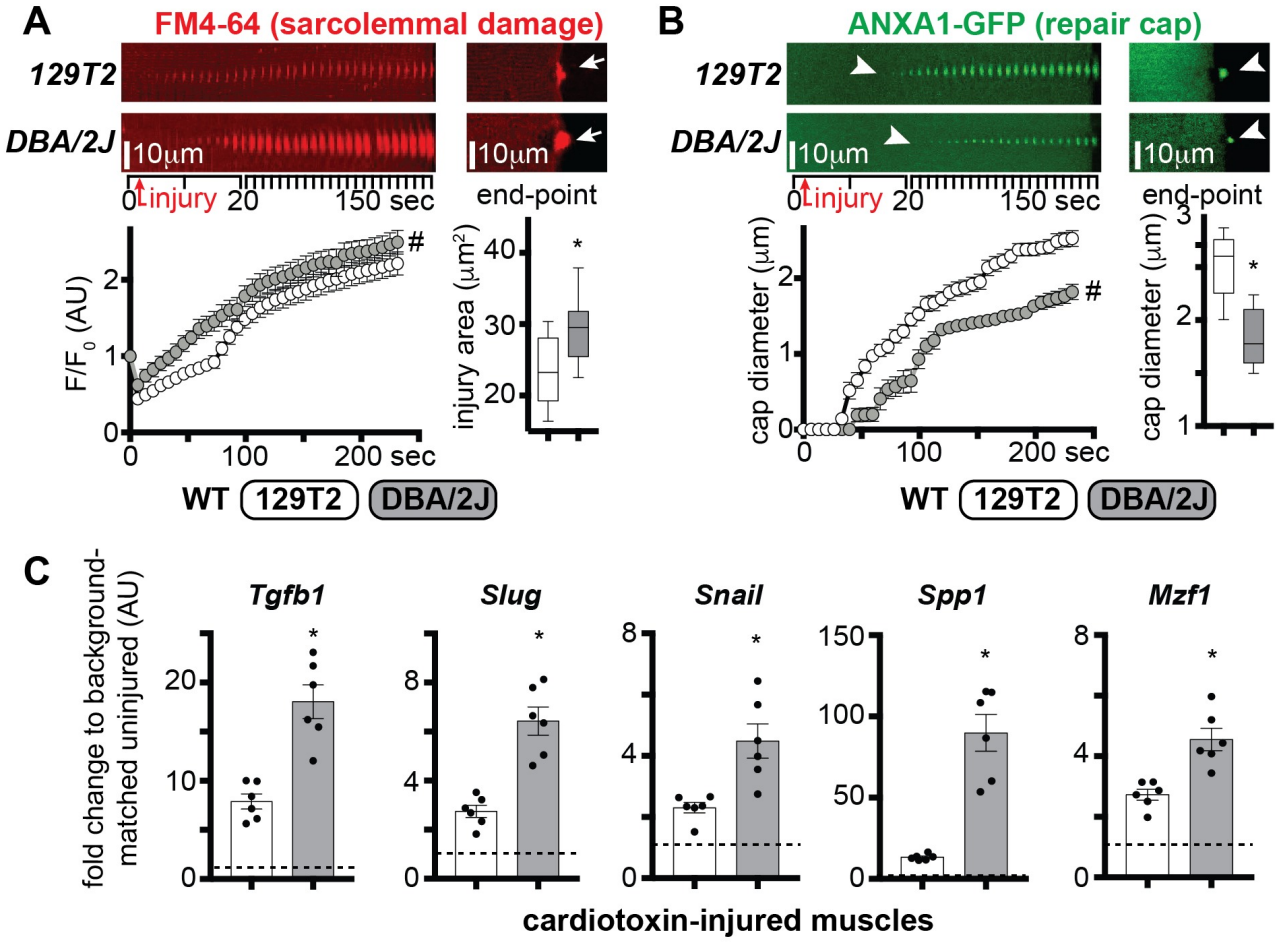
The *DBA/2J* genetic background is associated with increased sarcolemmal damage and higher levels of OPN and TGFβ pathway genes. The DBA/2J background exacerbates the muscular dystrophy phenotype [6, 9]. **A)** The extent of sarcolemmal damage was significantly higher in WT *DBA/2J* myofibers, as compared to age-matched WT *129T2* myofibers, both over time and at end-point (arrows). **B)** Repair cap formation, monitored by GFP-tagged ANXA1, appeared significantly delayed in *DBA/2J* myofibers (arrowheads), with a smaller cap diameter at end-point (arrowheads). The time series shows stacked consecutive images of the injured site at 10° orientation to reveal the extent of dye accumulation or repair cap formation. FM4-64 (red) and ANXA1-GFP (green) pictures were acquired simultaneously. **C)** qPCR analysis of cardiotoxin-injured *tibialis anterior* muscle tissue at 3 days post-injury from *129T2* and *DBA/2J* mice versus strain-matched uninjured littermates showed that injury-associated upregulation of *Tgfb1*, *Slug*, *Snail*, *Spp1*, and *Mzf1* was significantly higher in the *DBA/2J* strain than in the *129T2* strain (dotted line: strain-matched control values, = 1). Marked line plots, avg±sem; box plots, Tukey distribution; histograms, single values & avg±sem; n = 5 mice/group; #, P<0.05 vs vehicle, 2way ANOVA + Bonferroni; *, P<0.05 vs 129T2, unpaired t-test with Welch’s correction.

In *Sgcg* (γ-sarcoglycan) or *DMD* (*mdx*) mice, the *DBA/2J* background exacerbates the dystrophic phenotype [6, 9]. We sought to determine whether the difference in global outcomes associated with those genetic backgrounds was correlated with the efficiency of sarcolemma repair. To exclude effects associated with dystrophic remodeling, we tested WT mice from 129T2 and DBA/2J background using laser-induced sarcolemmal injury. DBA/2J myofibers showed more severe sarcolemmal damage than 129T2 myofibers (Fig 6A). Annexin A1 cap formation, seen as ANXA1-GFP, appeared more slowly in DBA/2J than in 129T2 myofibers and resulted in smaller cap size at end-point (Fig 6B).

Although many genetic loci contribute to the DBA/2J background effect, DBA/2J mice feature the *Ltbp4* risk allele, which is estimated to contribute to at least 40% of the variance of the muscular dystrophy phenotype in *Sgcg* mice [42]. RNA sequencing of WT and *Sgcg* muscle from the severe DBA/2J background was associated with a marked increase in *Spp1* expression compared expression in the 129T2 background (S3 Fig). We monitored gene expression changes relevant to the TGFβ pathway three days after cardiotoxin-mediated injury to the TA muscles of age-matched 129T2 or DBA/2J in order to assess the effect of genetic background. After muscle injury, the DBA/2J background was associated with increased expression of *Tgfb1*, *Slug*, *Snail*, *Spp1*, and *Mzf1* compared to injured 129T2 muscle (Fig 6C). Thus, the DBA/2J genetic background associated with increased sarcolemmal damage, and higher levels of osteopontin and TGFβ pathway genes.

### *LTBP4* modifies sarcolemmal repair of dystrophic myofibers

**Fig 7:**
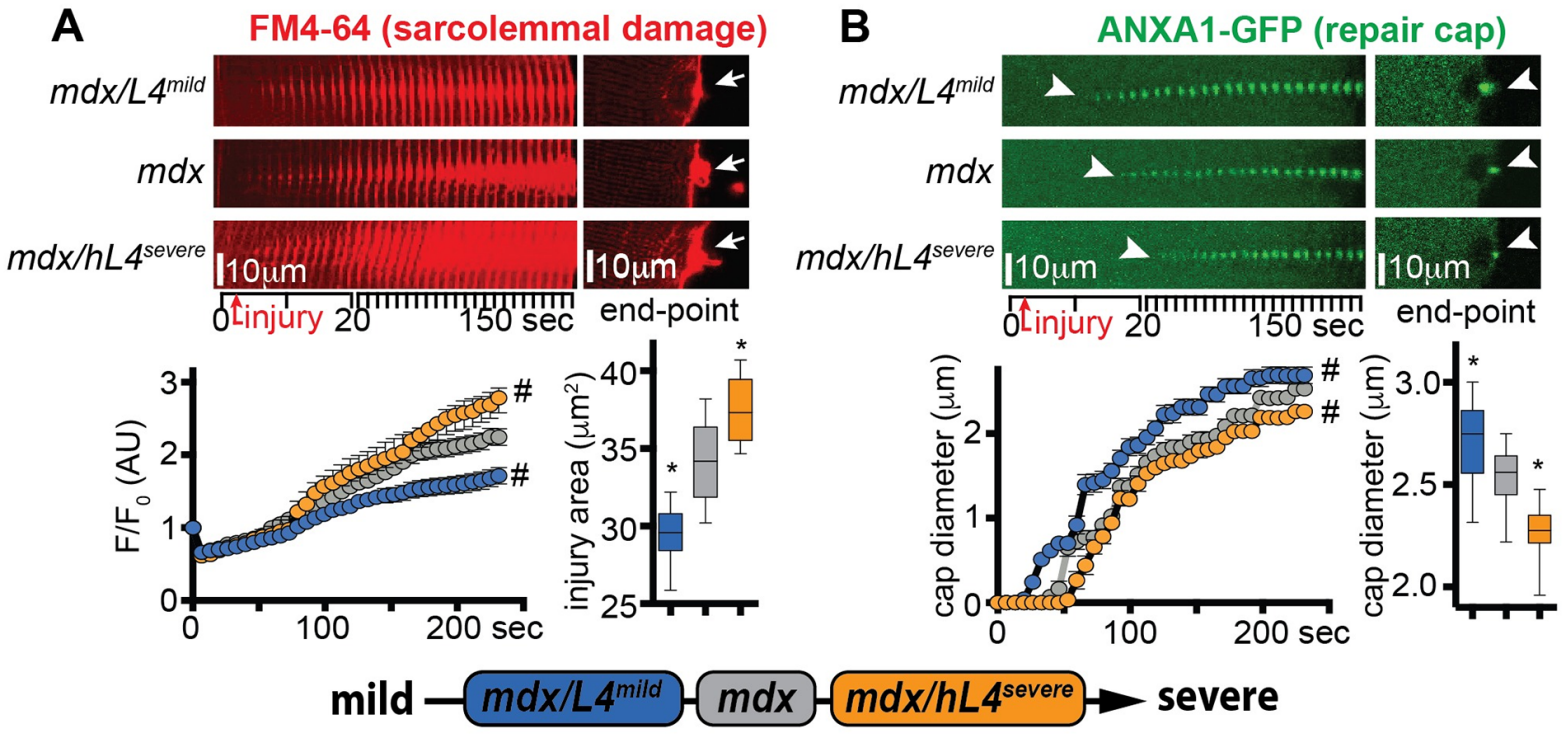
Disease-modifying LTBP4 isoforms differentially modify sarcolemmal repair in dystrophic muscle. Overexpression of the protective L4^mild^ allele in *mdx* mice (*mdx/L4*^*mild*^) protects against many features of muscular dystrophy [6]. Expression of the deleterious human *LTBP4* allele in *mdx* mice (*mdx/hL4*^*severe*^) worsens muscular dystrophy [44]. **A)** Sarcolemmal damage was significantly enhanced in *mdx/hL4*^*severe*^ myofibers, as compared to littermate, background-matched *mdx* myofibers, both over time and at end-point (arrows). Conversely, sarcolemmal damage was reduced in age-matched *mdx/L4*^*mild*^ myofibers. **B)** Repair cap formation, monitored by GFP-tagged ANXA1, was significantly delayed in *mdx/hL4*^*severe*^ myofibers (arrowheads), with a smaller cap diameter at end-point (arrowheads). Opposite trends were quantitated in age-matched *mdx/L4*^*mild*^ myofibers (arrowheads). Time series of stacked consecutive images of the injured site at 10° orientation revealed the extent of dye accumulation or repair cap formation. FM4-64 and ANXA1-GFP pictures were acquired simultaneously. Marked line plots, avg±sem; box plots, Tukey distribution; n = 50 myofibers (5 mice)/group; #, P<0.05 vs *mdx*, 2way ANOVA + Bonferroni; *, P<0.05 vs *mdx*, 1way ANOVA + Bonferroni.

Muscle specific overexpression of the protective *Ltbp4* allele (L4^mild^) in *mdx* mice reduces fibrosis and increases muscle size [43]. Conversely, expression of the risk human *LTBP4* allele (hL4^severe^) exacerbates dystrophic remodeling in *mdx* mice [44]. We tested sarcolemmal damage and repair cap formation in myofibers from transgenic age-matched *mdx* mice overexpressing either the L4^mild^ (*mdx/L4*^*mild*^), or the hL4^severe^ isoform (*mdx/hL4*^*severe*^) at 20 weeks of age. After laser-mediated sarcolemmal injury, the extent of sarcolemmal injury was smaller in myofibers of *mdx/L4*^*mild*^ mice than from *mdx* control animals. Conversely, sarcolemmal damage was increased in *mdx/hL4*^*severe*^ myofibers as compared to control muscles (Fig 7A). Repair cap formation, monitored through GFP-labelled annexin A1 (ANXA1-GFP), appeared faster in *mdx/L4*^*mild*^ and slower in *mdx/hL4*^*severe*^ myofibers than in *mdx* control myofibers. These trends associated with a bigger cap size in *mdx/L4*^*mild*^ and a smaller cap size in *mdx/hL4*^*severe*^, when compared to *mdx* repair caps at end-point (Fig 7B). Thus, the protective isoform with less TGFβ release associated with reduced sarcolemmal damage and faster cap formation, while the proteolysis-prone, risk allele with higher TGFβ release associated with increased damage and delayed repair cap assembly.

### Dissecting the effects of mild and severe alleles of *Anxa6* and *Ltbp4* on sarcolemmal repair and muscle injury in vivo

**Fig 8:**
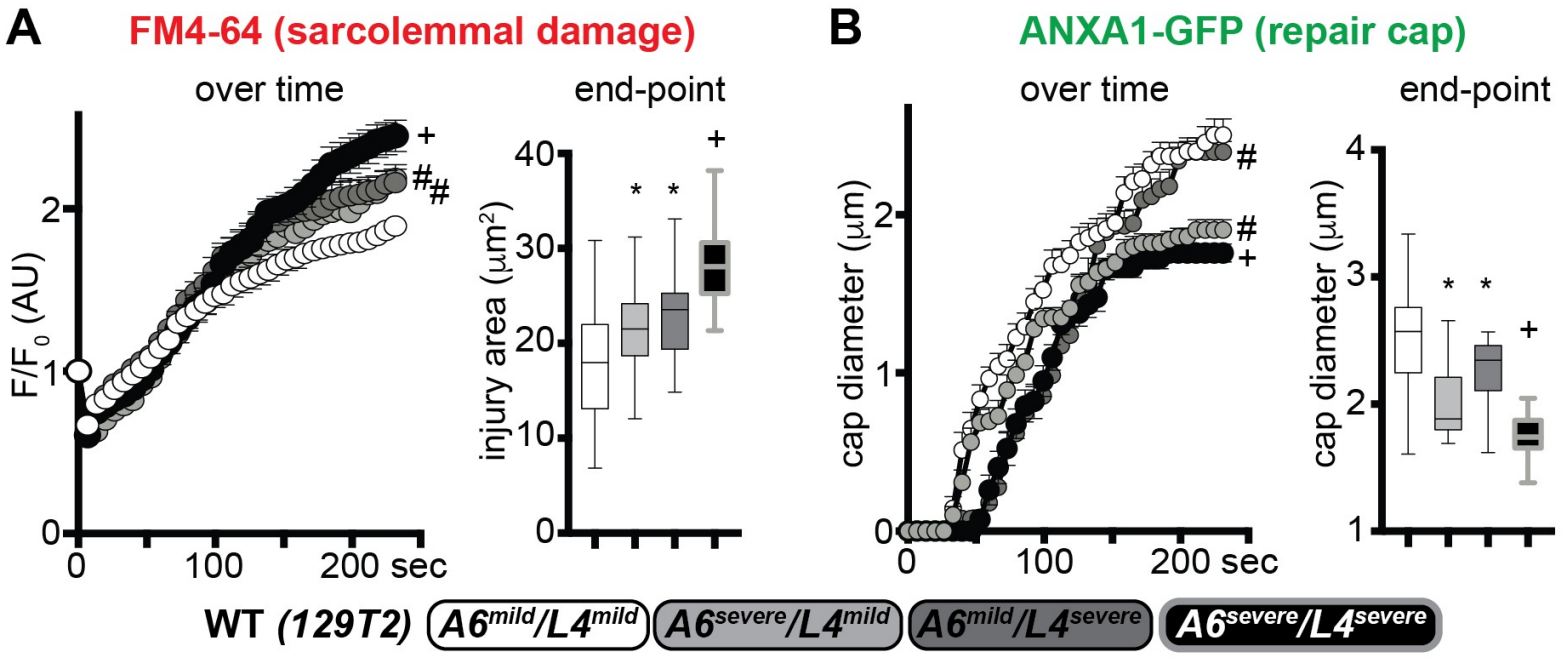
Relative contribution of *Anxa6* and *Ltbp4* alleles on sarcolemmal repair in wildtype muscle. In addition to *Ltbp4*, Anxa6 has also been shown to modify muscular dystrophy in mice [25, 45]. The deleterious alleles of *Ltbp4* (L4) or *Anxa6* (A6) (severe) were compared to those from the protective 129 strain (mild) in the sarcolemma injury assay. **A)** Doubly homozygous A6^mild^/L4^mild^ myofibers had the least injury while doubly homozygous A6^severe^/L4^severe^ fibers had the greatest injury, marked by FM4-64. Myofibers with mixed homozygous genotypes were intermediate with respect to FM4-64 marked injury. **B)** A similar pattern was observed for Annexin A1 (ANXA1) repair caps, where doubly homozygous mild alleles of L4 and A6 assembled caps more rapidly and produced larger repair caps than doubly homozygous severe alleles. However, ANXA1 repair caps were smaller with the A6 homozygous severe allele, despite the presence of the mild L4 allele, suggesting that repair cap formation is dominated by the A6 genotype. FM4-64 and ANXA1-GFP images used for the analyses in **A-B** were acquired simultaneously. Marked line plots, avg±sem; box plots, Tukey distribution; n = 50 myofibers (5 mice)/group; marked line plots: #, P<0.05 vs control (A6^mild^/L4^mild^), +, P<0.05 vs A6^severe^/L4^mild^ and A6^mild^/L4^severe^ groups, 2way ANOVA + Bonferroni; boxplots: *, P<0.05 vs control (A6^mild^/L4^mild^), +, P<0.05 vs A6^severe^/L4^mild^ and A6^mild^/L4^severe^ groups, 1way ANOVA + Bonferroni.

*Anxa6*, was also identified as a modifier of muscular dystrophy by its action on sarcolemmal repair itself [45], and the DBA/2J background harbors the risk allele for *Anxa6*. In order to discriminate the effects elicited by the *Anxa6* and *Ltbp4* genetic modifiers on sarcolemmal repair, we generated WT mice in the 129T2 background strain carrying the four homozygous combinations of mild and severe isoforms of *Anxa6* and *Ltbp4* (A6^mild^/L4^mild^; A6^severe^/L4^mild^; A6^mild^/L4^severe^; A6^severe^/L4^severe^). We assessed sarcolemmal damage and annexin A1 cap formation after laser injury in myofibers from age-matched mice from the four cohorts. Muscles homozygous for either A6^severe^ or L4^severe^ had increased sarcolemmal damage, when compared to A6^mild^/L4^mild^ myofibers. Moreover, the effect of the two severe isoforms appeared additive on the sarcolemmal damage phenotype, as A6^severe^/L4^severe^ myofibers presented the highest levels of FM4-64 accumulation across the groups (Fig 8A). Repair cap formation was also regulated by the additive effects of the severe isoforms. The A6^severe^ isoform associated with smaller cap size at end-point, while the L4^severe^ isoform associated with a delay in initial onset of formation, when compared to the A6^mild^/L4^mild^ genotype. Similarly, the A6^severe^/L4^severe^ correlated with onset delay and smaller size of the ANXA1 repair cap at end-point (Fig 8B). Thus, the polymorphism affecting LTBP4 function modifies sarcolemmal damage and repair cap formation in WT myofibers, and its effects are additive with respect to the *Anxa6* polymorphism.

**Fig 9:**
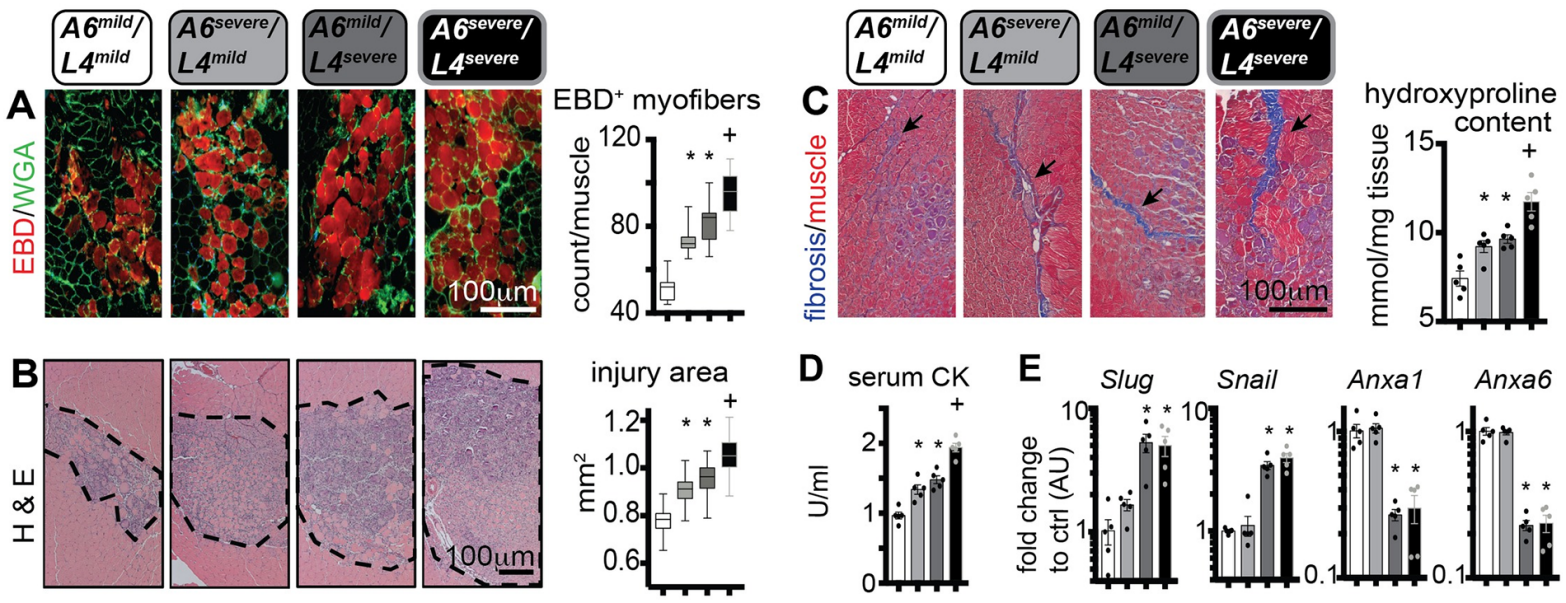
Relative contribution of *Anxa6* and *Ltbp4* alleles on muscle injury in vivo. **A)** Three days post-injury, the number of dye^+^ myofibers (red) in injured *tibialis anterior* muscles was increased in single homozygous mice, either A6^severe^ or L4^severe^, compared to double homozygous A6^mild^/L4^mild^ muscle, which displayed the fewest dye+ fibers. Doubly homozygous animals A6^severe^/L4^severe^ had the greatest number of dye^+^ fibers. **B)** The same pattern was seen when measuring injury area from cardiotoxin injected gastrocnemius muscles measured 7 days post-injury. **C)** Scarring monitored by Masson’s trichrome staining (left panels; arrows) and hydroxyproline content (right chart) was also greatest in doubly mutant (A6^severe^/L4^severe^*) gastrocnemius* muscles 7 days post-injury. **D)** 24 hours after cardiotoxin-injury of hindlimb muscles, serum CK levels were increased in single homozygous mice A6^severe^ or L4^severe^, and further increased in double homozygous A6^severe^/L4^sever*e*^ animals. **E)** Seven days after cardiotoxin injury, *Slug*, *Snail* were upregulated while *Anxa1*, and *Anxa6* expression levels were significantly downregulated in muscle in the presence of homozygous L4^severe^ allele (*tibialis anterior*) indicating that the L4^severe^ allele impacts *Anxa1/Anxa6* expression likely via *Slug/Snail* regulation. *, P<0.05 vs A6^mild^/L4^mild^, +, P<0.05 vs A6^severe^/L4^mild^ and A6^mild^/L4^severe^ groups, 1way ANOVA + Bonferroni.

We next subjected mice from all four allele combinations to intra-muscular injury with cardiotoxin, targeting both TA and *gastrocnemius* muscles. To quantify the number of injured myofibers, mice were intra-peritoneally injected with EBD immediately before intramuscular toxin injection. Three hours after dye delivery, the number of dye-positive myofibers in injured muscles was comparably higher in mice with one severe allele, and the highest in mice with both severe alleles, with respect to A6^mild^/L4^mild^ mice (Fig 9A). Seven days after injury, the injury area at muscle mid-point and fibrotic scarring followed similar trends, as quantified by histologic analyses (Fig 9B and 9C). Serum creatine kinase (CK) at 24 hours after injury was higher in the presence of either A6^severe^, or L4^severe^ alleles, and the highest in mice with both severe alleles, as compared to A6^mild^/L4^mild^ mice (Fig 9D). Seven days after injury, muscle tissue was analyzed for gene expression trends. Transcriptional levels of *Slug* and *Snail* were significantly upregulated in the presence of the L4^severe^ allele regardless of the A6 allele status. Expression of *Anxa1* and *Anxa6* was also downregulated in the presence of L4^severe^ isoforms (Fig 9E). Thus, *Anxa6* and *Ltbp4* isoforms modify the extent of damage in acute muscle injury in an additive fashion, and the severe isoform of *Ltbp4*, characteristic of the DBA/2J genetic background, correlated with upregulation of *Slug* and *Snail*, and downregulation of *Anxa1* and *Anxa6*.

## Discussion

### A feed forward loop between TGFβ and osteopontin in muscular dystrophy and muscle injury

Genetic modifiers of muscular dystrophy influence outcome through multiple pathways. However, their combinatorial effects, and specifically whether they are additive, synergistic or even opposing in action is challenging to address at a human population level in a rare disorder. Here we utilized a surrogate endpoint, sarcolemmal repair, to begin to assess how osteopontin and LTBP4 modify myofiber repair through a convergent TGFβ pathway circuitry. Furthermore, we showed that upregulated OPN/TGFβ results in transcriptional repression of annexin genes, and this provides one possible means by which the OPN/TGFβ pathway contributes to impaired sarcolemmal resealing. Given the broad gene expression effects of the OPN/TGFβ pathway, we expect that multiple genes mediate the in vivo effect in muscular dystrophy. In vivo, in the absence of osteopontin, *mdx* muscle had fewer disrupted myofibers, which likely reflected this feed forward loop altering sarcolemmal stability, repair, and even muscle regeneration.

Osteopontin and TGFβ signaling constitute a common marker of dystrophic muscle when compared to healthy controls [11–19, 46–49]. Conversely, genetic manipulation to stifle both cascades reduces dystrophic pathology [13, 36, 43, 50]. To date, these signaling pathways have been mainly associated with activation and tissue remodeling by immune cell infiltrates and resident fibroblasts [10, 51]. These results indicate that osteopontin and TGFβ pathways act synergistically to directly influence the ability of myofibers to repair after sarcolemmal injury. There is an expected crosstalk among myofibers, fibroblasts, and immune cells during both acute and chronic injury, which further modifies dystrophic features.

We found that the presence of either osteopontin or the deleterious form of *Ltbp4* resulted in annexin gene repression and increased sarcolemmal damage. A limitation of the sarcolemmal repair assays used for this study is the use of electroporation to express GFP-tagged annexins in the presence of native annexins. To address this, we relied on electroporation of the same construct (ANXA1-GFP) in all different genetic contexts, and the results observed in the sarcolemmal repair assay paralleled the results seen when examining muscle injury in vivo, in both dystrophic and WT settings. Importantly, sarcolemmal repair assays were conducted in the presence of Ca^2+^, as annexin repair cap formation is known to be Ca^2+^-dependent [32]. Further analyses in Ca^2+^-free settings, in combination with finer characterization of membrane composition, may discriminate *Spp1*- and *Ltbp4*-dependent effects on repair capacity and membrane mechanical stability, as recently investigated for the repair protein MG53 [52].

In both genetic settings, either *Spp1* ablation or the deleterious *Ltbp4* alleles, transcriptional regulation of *Anxa1 and Anxa6* inversely correlated with expression levels of *Slug/Snail* and occupancy of the TGFβ-related SLUG/SNAIL repressive complex on the E-box elements upstream of *Anxa1 and Anxa6*. These findings corroborate the hypothesis that both genetic modifiers contribute to a self-reinforcing, TGFβ-reliant loop in muscle, consistent with traditional observations of a feed-forward regulation of TGFβ signaling [37, 38]. The deleterious VTTT haplotype in the *LTBP4* gene correlated with more rapid disease progression in DMD patients, while the rs28357094 polymorphism in the *SPP1* promoter has been reported as a disease determinant in some studies, but not in others [19, 21, 22, 24, 53]. However, it is still unclear whether the combination of deleterious polymorphisms at both loci significantly associates with worsened disease outcome. Given the rare nature of DMD, assembling sufficient cohorts for these genetic studies in humans may not be possible.

### Insights on sarcolemmal stability and repair

Genome-wide screening for disease traits identified both *Ltbp4* and *Anxa6* as sarcolemmal damage modifying genes. The DBA/2J mouse strain carries both risk alleles and produces enhanced muscular dystrophy pathology in mice [25, 45]. *Anxa6* alleles regulate annexin repair cap size. However, LTBP4 content determines the efficiency of cap formation after injury, with the deleterious *Ltbp4* alleles associated with delayed cap formation. These detrimental effects were additive in the presence of both alleles, and translated in additive effects on the in vivo response to muscle injury. In addition to the transcriptional regulation of annexin genes, it is possible that LTBP4-mediated TGFβ overload impacts sarcolemmal repair through post-transcriptional regulation and/or protein interactions. *Ltbp4* genotype also appears to be a modifier of the extent of injury, which may reflect enhanced stability of the myofiber prior to injury.

The protective *LTBP4* allele in humans has been associated with delayed loss of ambulation in humans with DMD, and this effect was greater in the presence of glucocorticoid steroid regimen in those study cohorts [24]. Interestingly, steroid-associated additive beneficial effects were also reported for the *SPP1* polymorphism in DMD patients [21]. We recently reported that glucocorticoid steroids, such as prednisone and deflazacort, improve sarcolemmal repair and annexin cap formation in normal and dystrophic muscle [54]. It has been suggested that glucocorticoid steroids act in muscular dystrophy by synchronizing an asynchronous repair milieu in muscular dystrophy [55]. These data suggest that genetic modifiers beyond *SPP*1/OPN may contribute to this disorganized repair process and support the development of agents to resynchronize this process.

In summary, we now show that both osteopontin and LTBP4 directly modify sarcolemmal repair in myofibers of normal and dystrophic muscles. In the model, excess osteopontin and deleterious LTBP4 converge to sustain TGFβ-mediated gene expression changes, including the repression of annexins, and likely, many other repair genes. These findings indicate a direct role of those genetic modifiers in myofiber damage regulation and support their consideration for novel therapeutic avenues for treating dystrophic muscle.

## Materials and methods

### Ethics statement

Mice were housed in a specific pathogen free facility in accordance with Institutional Animal Care and Use Committee (IACUC) regulations. Euthanasia was performed through carbon dioxide or anesthetic gas inhalation followed by cervical dislocation and removal of the heart. All methods using living animals in this study were performed in ethical accordance with the American Veterinary Medical Association (AVMA) and under protocols fully approved by both the Institutional Animal Care and Use Committee (IACUC) at Northwestern University Feinberg School of Medicine (protocol number ISO00000911). Consistent with the approvals stipulated by these protocols, all efforts were made to minimize suffering.

### Animals

*mdx and mdx/Spp1*^*-/-*^ littermates from a mixed *BL/6-BL/10* background were previously described [13]. *129T2/SvEmsJ* (129T2) and *DBA/2J* WT inbred mice were purchased from the Jackson Laboratory (Bar Harbor, ME; Stock # 002065 and 000671, respectively). *Anxa6* and *Ltbp4* alleles from *DBA*/*2J* background were bred on the *129T2* background by means of initial *129T2* x *DBA/2J* breeding, followed by seven generations of mating the compound heterozygotes with *129T2* mice. All mice used for experiments with *DBA/2J Anxa6* and *Ltbp4* alleles were conducted on littermates obtained from mating pairs of compound heterozygotes from the *129T2* background. *Mdx* mice bearing the *BAC-hLTBP4* transgene or the *HSA*::*Ltbp4*^*129*^ transgene were previously described [43, 44]. Both *mdx* transgenic lines were generated and bred on a mixed *BL/6-BL/10* genetic background; the control cohort of mice for the experiments was created by pooling transgene-deficient littermates from both transgenic lines. Both females and males were used in *129T2* and *DBA/2J* mice experiments, while only males in experiments with *mdx* mice. Age of mice at the time of experiment was 8 weeks, unless otherwise specified. Mice were maintained on a 12 hour light/dark cycle and fed *ad libitum*.

### Plasmids

The plasmid encoding human annexin A1 with a carboxy-terminal GFP was obtained from Origene (Rockville, MD; Cat# RG201569).

### Electroporation, myofiber isolation, and laser injury

Myofibers from the flexor digitorum brevis (FDB) muscles were electroporated *in vivo*, as previously described in [56] with modifications described in [31]. Briefly, the footpad was injected with 10μl of hyaluronidase (8units) (Cat #H4272, Sigma, St. Louis, MO). After 2 hours, the footpad was injected with 20μl of 2μg/μl endotoxin-free plasmid. Electroporation was conducted with following parameters: 20 pulses, 20 ms in duration/each, at 1Hz, at 100 V/cm. Recovery was allowed for seven days after electroporation to avoid electroporation-induced damage and to allow plasmid expression [57]. Individual myofibers were then explanted and isolated as previously detailed [31].

Live myofibers were ablated with a laser as described [31, 32, 45]. Briefly, myofibers were dissociated in PBS supplemented with 0.2% BSA and 4mg/ml collagenase type II (Cat # 17101, Life Technologies, Grand Island, NY) at 37 degrees in 10% CO_2_. Muscle was triturated (20–30 pipetting motions through edge-cut 1000μl filter tip) after 60 and 120 minutes. Fibers were then seeded on MatTek dishes (Cat # P35G-1.5-14-C, MatTek, Ashland MA) in Ringers solution and, after 30 minutes, prepared for imaging by adding FM 4–64 dye (T-13320, Molecular Probes, Grand Island, NY) to a final concentration of 2.5μm.

Laser ablation and subsequent real-time imaging were performed at room temperature using a Nikon A1R laser scanning confocal equipped with GaSP detectors through a 60x Apo lambda 1.4 NA objective driven by Nikon Elements AR software. A single pixel set as 120 nm (0.0144 μm^2^) was ablated using the 405 nm laser at 100% power for up to 5 seconds. Images were acquired as follows: one image prior to damage (0 seconds; reference for relative fluorescence analyses), one image right after laser injury (bleach point), 10 images every 2 seconds after injury, and then one image every 10 seconds for up to 240 seconds after injury. Quality control for myofibers selected for laser ablation relied on following parameters. Only myofibers adherent to the MatTek dish from end to end and not contracting during imaging were used. Imaged fibers were required to have intact sarcomeres and unruptured sarcolemma. The region of the myofiber selected for laser injury was required to be linear without visible deformation or peripheral nuclei. ANXA1-GFP fluorescence within the myofiber body at time 0 was required to be between 200 and 2000 relative light units (RLUs), as per ImageJ analyses.

Relative fluorescence from an 85μm^2^ circular region encompassing the lesion area and ANXA1 cap diameter (perpendicular to myofiber axis) over time were calculated from images acquired as described above (FM4-64 and ANXA1 images acquired simultaneously), normalizing values to the pre-injury intensity. This method allows inter-group comparisons and reduces variability. Prism Graphpad was used to calculate averages, and values were normalized to the pre-bleach intensity (F/F_0_). All measurements were from n = 4 or 5 mice per group (depending on experiment), with ≥10 myofibers per mouse (total 40–50 per genotype or condition). Data analysis was conducted blinded to genotype/treatment group. Stack rendering of time course image sequences was conducted by using the “3D project” built-in feature of Image J with default parameters (Projection method: Brightest Point; Slice spacing: 1.00μm). Stacks were then tilted by 10° in order to show extent of sarcolemmal damage or repair cap formation over time.

### Recombinant osteopontin (rOPN) and chemical compound treatments

rOPN was purchased as lyophilized powder (Cat #441-OP; R&D Systems, Minneapolis, MN), resuspended as per manufacturer’s instructions, and stored at -80°C. Treatment of FDB myofibers with rOPN was conducted injecting the footpad with 10μg rOPN in 10μl PBS at 1, 3, and 5 days after electroporation. Laser injury was conducted seven days after electroporation. SB431542 and PF573228 (Cat # S4317 and PZ0117, respectively; Sigma-Aldrich; St. Louis, MO) were resuspended in DMSO (Cat #D2650; Sigma-Aldrich; St. Louis, MO) and stored at -20°C. C2C12 myoblasts (ATCC #CRL-1772; Manassas, VA; all experiments performed at passages 5–15 after ATCC batch thawing) were cultured in DMEM supplemented with 10% FBS (Cat #2442 (lot #14E332; Sigma, St. Louis, MO) 1% P/S (Cat #15070; Thermo Fisher Scientific, Waltham, MA). On treatment start day, myoblasts at ~40% confluence were treated with different protein/compound combinations, or with equal amounts of vehicle, as diluted supplement in growth medium. Myoblasts were harvested for qPCR of ChIP-qPCR analyses 48 hours after treatment onset. Chemical compounds were used at a final concentration of 10μM, while rOPN was diluted to a final concentration of 1μg/ml [40, 58]. Effectiveness of dosing in cells and muscles was tested in pilot experiments by means of qPCR analysis of *Slug*, *Snail*, and *Mzf1* mRNA at 24 hours after treatment onset. Eight independent replicates of C2C12 treatment groups were used for analyses. Analysis was conducted blinded to treatment group.

### Muscle injury and Evans-Blue Dye (dye) staining

Cardiotoxin injury was performed by injecting 20μl of a 10μM cardiotoxin (Cat #TXL1376-1; Accurate Chemical & Scientific Corporation, Westbury, NY) solution in PBS in target muscles in sedated animals (3% isoflurane, 0.8 l/min O_2_). Cardiotoxin was injected bilaterally in both *tibialis anterior* and *gastrocnemius* muscles. Cardiotoxin was released in the center of the muscle through the whole major axis, in order to have a homogenous area of injury at the center of the muscle. EBD staining (10mg/ml in PBS, sterile filtered; cat #E2129; Sigma-Aldrich; St. Louis, MO) was injected in a dedicated cohort of toxin-injured mice intra-peritoneally immediately before toxin-injury. Muscles were collected for fluorescence microscopic analysis 3 hours after toxin-injury.

### Serum collection and creatine kinase (CK) analysis

Serum was analyzed as previously reported [59] from animals 24 hours after toxin injury. Serum creatine kinase was analyzed in triplicates for each mouse using the EnzyChrom Creatine Kinase Assay (Cat # ECPK-100; BioAssay Systems, Hayward, CA) following manufacturer’s instructions. Synergy HTX multi-mode plate reader (BioTek^®^, Winooski, VT) was used to collect data, expressed as U/ml. Analysis was conducted blinded to treatment group.

### Histology, immunofluorescence microscopy (IF) and antibodies

Explanted muscles were fixed in 10% formaldehyde (Cat #245–684; Fisher Scientific, Waltham, MA) for histologic processing, or frozen in liquid nitrogen, inside pre-cooled Nalgene cryovials, and stored at -80°C for molecular analyses, or embedded in tissue freezing medium (Cat #TFM-5; Triangle Biomedical Sciences, Durham, NC) for IF analyses. Seven μm sections from the center of paraffin-embedded muscles were stained with hematoxylin and eosin (H&E; cat #12013B, 1070C; Newcomer Supply, Middleton, WI) and Masson’s trichrome (Cat #HT-15; Sigma-Aldrich; St. Louis, MO). Injury area was quantitated from >30 non-consecutive sections per muscle. Analyses were conducted blinded to treatment group. Ten μm sections from the center of frozen-embedded muscles were collected on the cryostat (chamber, -20°C; sample, -15°C; cat #CM1950; Leica, Wetzlar, Germany) for immunostaining. At least 30 non-consecutive sections were analyzed per muscle per condition. Analyses were conducted blinded to treatment group.

IF staining was performed using the following conditions: 4% PFA fixation (10 minutes, room temperature); permeabilization with 0.2% Triton (Cat #X-100; Sigma-Aldrich; St. Louis, MO), 1% bovine serum albumin (Cat #A7906; Sigma-Aldrich; St. Louis, MO) PBS (30 minutes, room temperature); blocking in 1% BSA, 10% FBS PBS (30 minutes at room temperature). For pSmad3^+^ myonuclei detection, rabbit polyclonal primary antibody (Cat #ab51451; Abcam, Cambridge, MA) staining was counterstained with 1μg/ml WGA conjugated to AlexaFluor594 (Cat #W11262; Thermo Fisher Scientific, Waltham, MA) at room temperature for 1 hour, to outline myofibers. For EBD^+^ myofiber detection, sections were counterstained with 1μg/ml WGA conjugated to AlexaFluor488 (Cat #W11261; Thermo Fisher Scientific, Waltham, MA) at room temperature for 1 hour; nuclei were counterstained with 0.5μg/ml Hoechst PBS (45 minutes, room temperature). EBD is spontaneously fluorescent in the TRITC channel. Imaging was performed using a Zeiss Axio Observer A1 microscope, using 10X and 20X objectives. Gryphax software (version 1.0.6.598; Jenoptik, Jena, Germany) was used for brightfield pictures, while ZEN 2 software (version 2011; Zeiss, Jena, Germany) was used for immunofluorescence images. Quantitation of injury area and myofiber count was based on sections collected throughout the major muscle axis (at least 10 sections per muscle per animal) and was performed using ImageJ (NIH).

### Hydroxyproline quantification

Frozen quadriceps muscles (100mg) was used to measure hydroxyproline content, as previously described [25]. Analyses were conducted blinded to treatment group. Results were reported as mmol (HOP)/mg (tissue).

### Quantitative RT-PCR

Total RNA was extracted by means of Trizol (Cat #15596018; Life Technologies, Grand Island, NY) from 30mg tissue as per manufacturer’s instructions. Reverse transcription used two μg of RNA with the qScript cDNA kit (Cat #95048; Quanta Biosciences, Beverly, MA) following kit’s instructions. cDNA was diluted 1:7 and 2μl was used per 10μl qPCR reaction. Each qPCR reaction contained 100nM primers and 5μl iTaq SybrGreen Mix (Cat #1725124; Bio-Rad, Hercules, CA). The list of primers and sequences is provided in S1 Table. CFX96 RealTime System (Bio-Rad, Hercules, CA) was used to run the qPCR reaction (95°C, 15sec; 59°C, 60sec; 40 cycles) and quantitate fluorescence. Relative fold change among biological groups was calculated using *Pgk* as internal normalizer.

### Chromatin immunoprecipitation (ChIP)-qPCR and luciferase assays

ChIP-qPCR was performed according to previously reported conditions [60] and adjustments [61]. Forty-eight hours after treatment, 10^6^ myoblasts were collected, washed, fixed in 1% PFA. Fixation was quenched with 0.1375 mmol glycine (Cat #G7126; Sigma-Aldrich; St. Louis, MO). After lysis of cells and nuclei, chromatin was sonicated for 15 cycles (30 sec, high power; 30 sec pause) in a water bath sonicator set at 4°C (Bioruptor 300; Diagenode, Denville, NJ). One μg chromatin was used for pull-down or for input control samples. The primary antibody (anti-SLUG/SNAIL, cat #ab180714, Abcam, Cambridge, MA) were added at a 1:100 dilution in 300μl final volume, while shaking overnight at 4°C. Chromatin complexes were precipitated with proteinA/G beads (cat #20421; Thermo Scientific, Waltham, MA). DNA was de-complexed with 0.07μg/μl proteinase K (cat #19131; Qiagen, Hilden, Germany) at 55°C and purified through QIAQuick PCR purification kit (cat #28106; Qiagen, Hilden, Germany). qPCR amplification was conducted as described for gene expression analysis, with a dedicated thermal profile (95°C, 30 sec; 55°C, 30 sec; 72°C, 30 sec; 50 cycles). Results were expressed as % of raw expression of the respective input. ChIP-qPCR on isolated myofibers was performed as above, with the following adjustments before chromatin sonication. Freshly-isolated *gastrocnemius* muscle was finely minced and digested in 5ml/muscle of PBS supplemented with 1mM CaCl_2_ and 100U/ml collagenase II (Cat # 17101, Life Technologies, Grand Island, NY) at 37°C for 1 hour with shaking. After filtration through a 40μm strainer (Cat # 22363547, Fisher Scientific, Waltham, MA), the unfiltered fraction (enriched in myofibers) was kept for further procedures. Separation of mononuclear fraction from myofibers in the filtered suspension was confirmed at a microscope. Myofiber lysis was performed in lysis buffer, using 700μl per muscle, with ~250μl 2.3mm zirconia/silica beads (Cat # 11079125z, BioSpec, Bartlesville, OK). Lysis buffer consisted of 10mM HEPES (pH 7.3; Cat # H3375), 10mM KCl (Cat # P9541), 5mM MgCl_2_ (Cat # M8266), 0.5mM DTT (Cat # 646563), 3μg/ml cytochalasin B (C6762; all reagents from Sigma, St. Louis, MO); protease inhibitor cocktail (Cat # 11852700, Roche, Mannheim, Germany)). Myofibers were homogenized at the Mini-BeadBeater-16 (Cat # 607, Biospec, Bartlesville, OK) for 30 sec, then by rotating at 4°C for 30 min. Samples were then centrifuged at 3000g for 5 minutes at 4°C; pellet was resuspended in cell lysis buffer, as per described protocol [60], supplemented with 3μg/ml cytochalasin B, and incubated on ice for 10 minutes. Nuclei were pelleted at 300g for 10 min at 4°C, and resuspended in 1ml 1% PFA for 5 min at room temperature. Fixation was quenched with 100μl of 1.375M glycine (Cat # BP381-5, Fisher Scientific, Waltham, MA). Nuclei were re-pelleted as before, and then processed following the described procedure [60], as mentioned for myoblasts. However, all solutions were supplemented with 3μg/ml cytochalasin B during chromatin preparation and sonication, antibody incubation, and wash steps.

### Statistical analysis and data presentation

Statistical analyses were performed with Prism (Graphpad, La Jolla, CA). Tests used for statistical comparison depended on group number and normality test (Pearson-D’Agostino). Typically, 2way ANOVA with Bonferroni multi-comparison was used to compare treatment or genotype effect on curves over time. When comparing >2 groups for one variable (typically treatment, or genotype), 1way ANOVA + Bonferroni multi-comparison was used. When comparing two groups for one variable, unpaired t-test with Welch’s correction was used, in order to account for skews in standard deviation between groups. For ANOVA and t-test analyses, a P value less than 0.05 was considered significant. Data were presented as single values (dot plots, histograms) when the number of data points was less than 10. In analyses pooling larger data point sets per group, Tukey distribution bars were used to emphasize data range distribution and histograms with error bars were used to emphasize shifts in average values. Analysis pooling data points over time were presented as marked line plots. Dot plots, histograms and marked line plots depict mean ± SEM. Box plots depict the Tukey distribution of the data pool: interquartile distribution; lower whisker, 25^th^ percentile minus 1.5 times the interquartile range; upper whisker, 75^th^ percentile plus 1.5 times the interquartile range.

## Supporting information

S1 Table List of primers for qPCR. (DOCX)

S1 Fig **A**) Seven days after injection of rOPN to FDB muscles, qPCR analysis shows upregulation of *Mmp2* and *Mzf1*:markers downstream of osteopontin-integrin signaling. Histograms, single values & avg±sem; n = 5 mice/group; *, P<0.05 vs vehicle, unpaired t-test with Welch’s correction. **B)** Diagram depicting the confocal imaging series used in these studies following laser injury to create sarcolemmal disruption. Image complication was used for both FM4-64 and ANXA1 image stacks over time.
(TIF)

S2 Fig mdx/*Spp1*^*-/-*^ muscles had increased annexins, reduced fibrosis and reduced CK compared to *mdx* muscles. **A)** Expression levels of *Anxa1* and *Anxa6* were upregulated in TA muscles of *mdx*/*Spp1*^*-/-*^ mice, as compared to control *mdx* animals. **B)** Hydroxyproline content, as an indicator of fibrosis, was reduced in *mdx/Spp1*^*-/-*^ quadriceps and diaphragm muscles compared to *mdx* muscles. **C)** Serum CK levels were significantly decreased in *mdx/Spp1*^*-/-*^ mice compared to control *mdx* animals. Histograms, single values & avg±sem; n = 4 mice/group; *, P<0.05 vs *mdx* control, unpaired t-test with Welch’s correction.
(TIF)

S3 Fig The *DBA/2J* background associates with higher levels of *Spp1* expression as compared to the *129T2* background, in both wildtype and dystrophic conditions. RNA-Seq analysis of quadriceps muscle tissue from *129T2-Sgcg*^*-/-*^ and *DBA/2J-Sgcg*^*-/-*^ mice versus strain-matched WT littermates. Fold change analysis showed that *Spp1* was upregulated in the presence of dystrophic remodeling in both strains. Moreover, Spp1 was consistently upregulated in *DBA/2J* muscle, as compared to the *129T2* muscle, in both wildtype and dystrophic conditions. Histograms, single values & avg±sem; n = 3 mice/group; *, P<0.05 vs designated group, 1way ANOVA + Bonferroni.
(TIF)
