# Supplementary material for: Genetic modifiers of muscular dystrophy act on sarcolemmal resealing and recovery from injury
Source: PLoS Genet. 2017 Oct 24;13(10):e1007070. doi: 10.1371/journal.pgen.1007070 (PMC5669489; doi:10.1371/journal.pgen.1007070)
Supplement: S1 Table — (DOCX) [file pgen.1007070.s001.docx]

**S1 Table. List of primers for qPCR.**

mmANXA6 Fw TGGCTGCTGAGATCTTGGAAA

mmANXA6 Rev CGTCCTTGACATCCCCAGAC

mmANXA1 Fw GGAGAAAGGGGACAGACGTG

mmANXA1 Rev TGGCACACTTCACGATGGTT

mmFBXO32 Fw TTGGATGAGAAAAGCGGCAG

mmFBXO32 Rev TACAGTATCCATGGCGCTCC

mmMSTN Fw CCCAGGACCAGGAGAAGATG

mmMSTN Rev AGTCCCATCCAAAGGCTTCA

mmMZF1 Fw AAGCTTCAGACTCCAGACCC

mmMZF1 Rev CGCTATGAGGAGAGGTCTGG

mmPGK Fw CAAAATGTCGCTTTCCAACAAG

mmPGK Rev AACGTTGAAGTCCACCCTCATC

mmSLUG Fw TTTCAACGCCTCCAAGAAGC

mmSLUG Rev AAAGGAGAGTGGAGTGGAGC

mmSNAIL Fw AAGCCCAACTATAGCGAGCT

mmSNAIL Rev TTTTGCCACTGTCCTCATCG

mmSPP1 Fw CCTGGCTGAATTCTGAGGGA

mmSPP1 Fw TATAGGATCTGGGTGCAGGC

mmTGFB1 Fw CAACTTCTGTCTGGGACCCT

mmTGFB1 Rev TAGTAGACGATGGGCAGTGG

mmTGFB2 Fw AGGATAATTGCTGCCTTCGC

mmTGFB2 Rev TGGTCAGTGGTTCCAGATCC

mmTGFB3 Fw CAGACACAACCCATAGCACG

mmTGFB3 Rev CTCAGCTGCACTTACACGAC

mmTGFBR1 Fw GAGAAGTTTGGCGAGGCAAA

mmTGFBR1 Rev ATCTGACACCAACCACAGCT

mmTGFBR2 Fw CGGGCGAGACTTTCTTCATG

mmTGFBR2 Rev GTGGACACGGTAGCAGTAGA

mmTWIST Fw GCCAGGTACATCGACTTCCT

mmTWIST Rev TCCAGCTCCAGAGTCTCTAGA
