# Supplementary figures and images for: Genetic modifiers of muscular dystrophy act on sarcolemmal resealing and recovery from injury
Source: PLoS Genet. 2017 Oct 24;13(10):e1007070. doi: 10.1371/journal.pgen.1007070 (PMC5669489; doi:10.1371/journal.pgen.1007070)

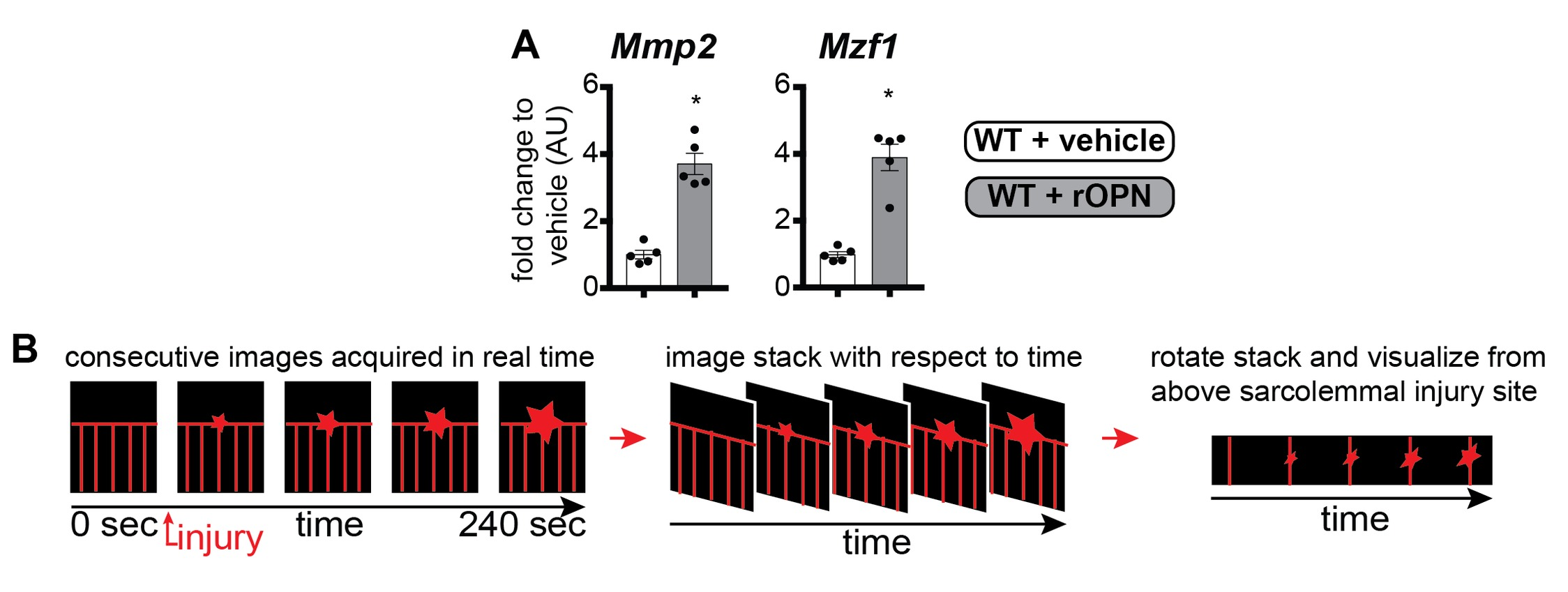

Supplement: S1 Fig — A) Seven days after injection of rOPN to FDB muscles, qPCR analysis shows upregulation of Mmp2 and Mzf1:markers downstream of osteopontin-integrin signaling. Histograms, single values & avg±sem; n = 5 mice/group; *, P<0.05 vs vehicle, unpaired t-test with Welch’s correction. B) Diagram depicting the confocal imaging series used in these studies following laser injury to create sarcolemmal disruption. Image complication was used for both FM4-64 and ANXA1 image stacks over time. (TIF) [file pgen.1007070.s002.tif]

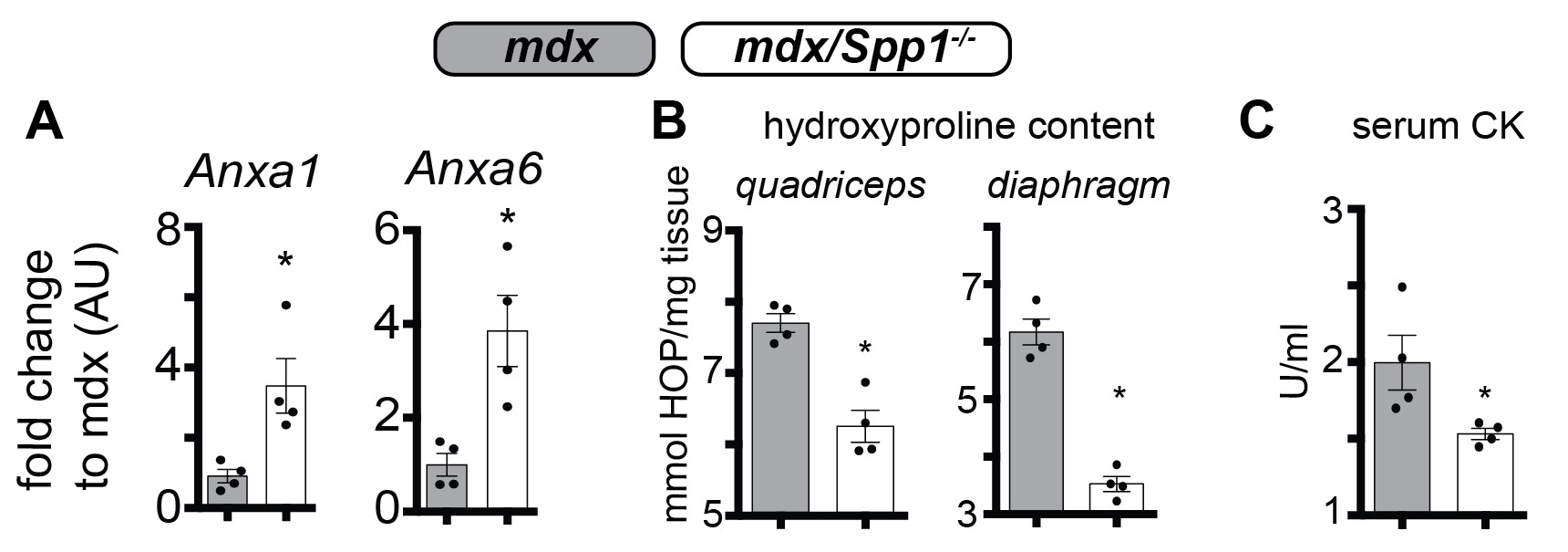

Supplement: S2 Fig — A) Expression levels of Anxa1 and Anxa6 were upregulated in TA muscles of mdx/Spp1-/- mice, as compared to control mdx animals. B) Hydroxyproline content, as an indicator of fibrosis, was reduced in mdx/Spp1-/- quadriceps and diaphragm muscles compared to mdx muscles. C) Serum CK levels were significantly decreased in mdx/Spp1-/- mice compared to control mdx animals. Histograms, single values & avg±sem; n = 4 mice/group; *, P<0.05 vs mdx control, unpaired t-test with Welch’s correction. (TIF) [file pgen.1007070.s003.tif]

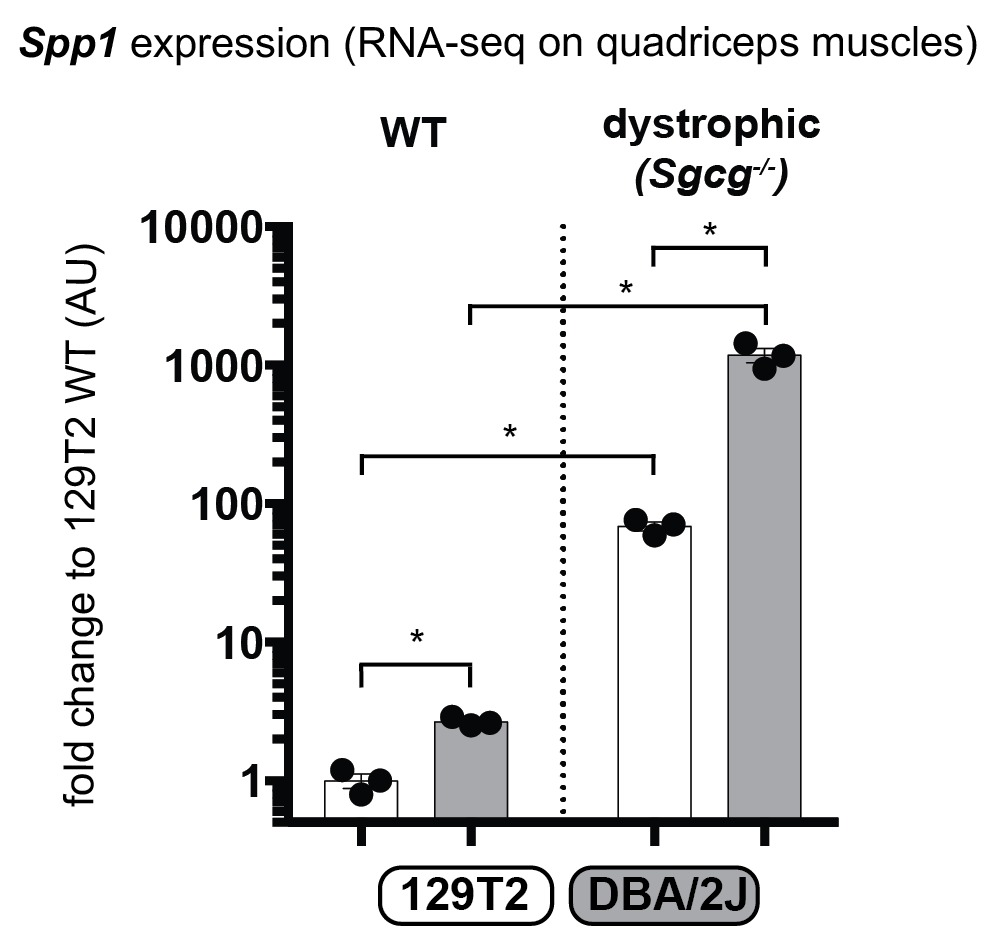

Supplement: S3 Fig — RNA-Seq analysis of quadriceps muscle tissue from 129T2-Sgcg-/- and DBA/2J-Sgcg-/- mice versus strain-matched WT littermates. Fold change analysis showed that Spp1 was upregulated in the presence of dystrophic remodeling in both strains. Moreover, Spp1 was consistently upregulated in DBA/2J muscle, as compared to the 129T2 muscle, in both wildtype and dystrophic conditions. Histograms, single values & avg±sem; n = 3 mice/group; *, P<0.05 vs designated group, 1way ANOVA + Bonferroni. (TIF) [file pgen.1007070.s004.tif]
